# A Comparative Analysis of Perceived Advantages and Disadvantages of Online Learning

**DOI:** 10.3390/bs13030262

**Published:** 2023-03-16

**Authors:** Marcel Pikhart, Blanka Klimova, Anna Cierniak-Emerych, Szymon Dziuba

**Affiliations:** 1 Faculty of Informatics and Management, University of Hradec Kralove, 500 03 Hradec Králové, Czech Republic; 2 Faculty of Informatics and Management, Wroclaw University of Economics and Business, 53-345 Wrocław, Poland

**Keywords:** applied linguistics, FLL, L2 acquisition

## Abstract

The use of electronic media has increased dramatically in the past decade due to the general increase in digitization of global societies. This trend has been recently enhanced by the COVID-19 occurrence and following forced implementation of various forms of eLearning into university curricula, including all forms of second language (L2) acquisition. The present study focuses on the evaluation of perceived advantages and disadvantages of online L2 acquisition via electronic media by university students of the Czech Republic (n = 114) and Poland (n = 121). The research methodology was an online questionnaire asking the users of digital media for L2 acquisition about their perceived advantages and disadvantages regarding the use of print and digital media and their potential impact on their L2 acquisition. To understand their evaluation is crucial as it could lead to increased motivation or demotivation to learn a foreign language. The results clearly show that the students realize the drawbacks of digital media and this could lead to their dissatisfaction and frustration when they have to use these media excessively. The implications of the findings could be helpful and necessary for various course designers, curricula makers, and course tutors as they are responsible for the smooth implementation of various digital tools into the educational process.

## 1. Introduction

Second language acquisition through various electronic media has been present since the very beginning when they were introduced [1,2,3]. Various forms of eLearning have been implemented and used at all levels of education and in their various stages, from the beginning (kindergartens and primary schools) to the late age (universities of the third age, U3A) [4,5]. eLearning itself has transformed significantly as it developed through different phases, from eLearning 1.0 through eLearning 2.0 and 3.0 up to now with the most current eLearning 4.0 with its utilization of artificial intelligence, big data, deep learning, machine learning, and other very recent trends in ICT [6,7,8,9].

The use of electronic media in L2 acquisition has brought many challenges as it is naturally beneficial but negative features can also be present [10,11,12,13], and the positive effects of L2 acquisition on human mental and cognitive abilities and well-being have already been well documented [14]. The negative effect of eLearning has not been studied sufficiently yet, despite the enormous literature about and research into the benefits of electronic media and various ICT platforms for L2 acquisition.

In addition, a subjective perspective, i.e., how the users of electronic media perceive the benefits or drawbacks of electronic media, particularly in the learning process of intentional L2 acquisition, has not been explored much either. This analysis, i.e., how it is perceived by the users of digital media, is very important as the content or discontent can create very strong feelings of satisfaction or dissatisfaction, or even excitement or frustration, and thus motivation or demotivation as one can perceive in sustainable multilingual education [15]. These feelings can be a great stimulus or deterrent having a large impact on an intentional L2 acquisition process and success [16,17,18]. It is very difficult for the users of various digital platforms to stick to very basic criteria necessary for the successful and impactful design of these tools, i.e., presenting material in their meaningful context, capitalizing on social affordances, and finally providing meaningful feedback to the participants [19].

The COVID-19 situation in years 2020–2022 made the implementation of digital media omnipresent in basically all European countries; therefore, many university students have a very wide experience with this enforced implementation. One of the popular online tools used in Poland and the Czech Republic in educational settings, including foreign language teaching and learning, is the Microsoft (MS) Teams platform. This platform is used widely for the following reasons, (compare with [20]):

- availability as an app on desktop computers and phones
- easy flexibility, user-friendliness of this online platform
- possibility to interact with students and provide immediate feedback
- possibility to create virtual classrooms in the form of teams
- possibility to share and edit documents and slides collaboratively
- opportunity to plan and run virtual meetings
- possibility to record and stream
- availability of testing students’ knowledge
- reduction of e-mail burden

The findings of research studies show that there is no consensus as far as the students’ satisfaction with learning via online platforms where L2 acquisition is concerned. Some students seem to be satisfied with this type of learning [21], while other students dislike this type of learning [22], which results mainly from participants´ digital illiteracy, worsening quality of education or technology costs [23], the lack of face-to-face interaction with the instructor, response time, and absence of traditional classroom socialization [24]. The rationale of the study is thus to find the answer to the question of whether the users of online classes consider them useful and beneficial for their L2 acquisition. Moreover, it also aims at collecting the drawbacks that could be related to this means of learning. Previous studies did not shed enough light on this question and this study attempts to answer the question.

The aim of this research is to determine how the users of online platforms for L2 acquisition perceive its benefits and drawbacks. To obtain reliable data, a large-scale questionnaire survey was conducted in two European countries, specifically in the Czech Republic and Poland, and factor analysis was conducted. Furthermore, the research questions were formulated as follows:

Q1: What will the respondents´ preference be regarding online classes conducted by MS Teams vs. face-to-face ones?
Q2: What kind of media will be preferred more for L2 acquisition, print media or digital media?
Q3: What are the main drawbacks of foreign language online learning when using MS Teams?

## 2. Materials and Methods

The research was conducted at the end of April and the beginning of May 2021 in two Central European universities, namely, a Czech and a Polish university, comparatively of the same size. It was the time when basically all university educational activity was conducted online, including language classes. All the participants had their English classes online via MS Teams. The time of the data collection was the end of the semester. Therefore, the students already had some experience and personal reflection of another semester of online classes.

The data were collected via an online questionnaire through Google Forms. The questionnaire was in English for both the Czech and Polish students as all of them study English and their knowledge of English is sufficient to understand without any problems and to be able to express what they want in English. The questionnaire consisted of two major parts: first, respondents’ data, containing 5 strictly sociodemographic questions and one filter question; and second, which was a core part, contained 19 statements using a standardized 5-point Likert scale (strongly disagree, disagree, undecided, agree, and strongly agree).

One multiple-choice question was included as well (Which language skill/s do you think are good to practice online?), and 3 open-ended questions to provide the respondents with an opportunity to freely express their opinion; however, they were only considered to be filler questions. Some of the key questions were doubled to verify the respondents’ previous answers but they were rephrased so that the participants were not able to realize they are basically the same. The respondents were not informed about the scope of the survey, on the other hand, they were told that the survey was conducted to improve their language courses.

The survey was performed with a total number of 235 respondents who all were university students from Poland (n = 121) and the Czech Republic (n = 114), at the Wroclaw University of Economics and Business and the University of Hradec Kralove. The students were students of ICT, management, financial management, and chemistry. As the research sample was large enough for this kind of research, it is statistically relevant and valid. Random sampling was used to select the participants.

The data were collected online, and all the respondents expressed their agreement with their participation in the survey by taking part in it, it was fully voluntary, and no instruction was given to them by the researchers. We did not collect any personal data about the participants and no email addresses or any other identification of the respondents. There were only a few demographic questions at the very beginning of the questionnaire, such as gender and the age of the respondent, so as to receive basic information about the participants. The only identification of the respondent was the date stamp of the particular questionnaire that only contained the time stamp when the questionnaire was finalized and submitted. No IP addresses were collected either. The study was also confirmed by the Committee for Research Ethics at the University of Hradec Králové, No. 2/2021.

Before starting the data analysis to obtain a statistically relevant initial research sample, the final form of the database was determined, based on the criteria as follows:

All duplicate records were removed from the Czech part.

Records of persons who indicated a nationality other than “Polish” or “Czech” were excluded from the analysis as well. The reason for this decision was the ambiguously interpretable answers to the question “What foreign language/s are you studying online?”.

Records of individuals who answered in the negative to the question “Do you have a foreign language online course (such as MS Teams) with your teacher every week during a semester at your university?” were excluded from the analysis because these individuals did not answer questions from the core section that followed after the initial section.

It was decided to include the aforementioned multiple-choice question from the core section into the respondent data section. A question, which is the sum of the indicated skills in the question “Which language skill/s do you think are good to practice online?”, was added to the respondent data section. A question was added to the metric section, which is the sum of the indicated foreign languages studied by the respondent. Eventually, after all these necessary exclusion criteria were executed, the database to be statistically analyzed consisted of 100 records of Czech nationality students and 107 records of Polish nationality students.

In the next step, it was decided to aggregate the following data:

Age: the raw database contained information about the respondent’s age, without any division into ranges. For readability of data interpretation, it was decided to aggregate the data into 4 ranges: A1—respondents aged 18 to 19 years, A2—respondents aged 20 years, A3—respondents aged 21 years, and A4—respondents aged 22 to 29 years.

Foreign languages: the raw database included information about which foreign language the respondent was learning. Due to the low number of indications for French, Italian, Russian, Danish, Japanese, and Portuguese, these languages were aggregated (other).

Foreign languages (number): due to the low number of indications of 3 or more languages (n = 1), it was decided to aggregate the data into 2 ranges: One—only one language was indicated, 2 and more—2 or more languages were indicated.

The largest number of students belonged to age group 3 (38.2%). The distribution between the first three age groups in the Polish study is almost even, while the fourth group included only one in ten Polish students. In the Czech group, the approximate number of indications is observed for A3 and A4. Almost every second Czech respondent was twenty years old (47.0%). Overall, the average age of respondents was 20.5 years (20.7 for CZ, 20.3 for PL), which makes it very similar and therefore the difference is statistically negligible. Slightly more men participated in the survey (52.7%), both in the Czech and Polish groups, but again, this discrepancy is statistically negligible.

All respondents studied English, with English being the only language studied by students in the Czech group for the most part (one language: 92.0%). The opposite situation is observed in the case of the Poles. Here, learning only one language was rare (1.9%). In addition to English, students from the Polish group most often studied German (42.1%) and Spanish (38.3%).

Both groups most often spent between 6 and 12 h in front of the computer (on working days: Monday–Friday), which included activities such as studying, entertainment, using social media, e-mails, etc. The skills indicated by Czechs were mainly listening skills (67.0%), vocabulary skills (66.0%), and speaking skills (50.0%), while in the case of Polish students, these were vocabulary skills (63.6%), reading skills (57.9%), and listening skills (55.1%). The largest differences in responses were found for writing skills (CZ < PL). Respondents most often limited themselves to indicating three or four skills (27.1% each). The details are in Table 1.

Due to the noticeable heterogeneity of the Czech and Polish groups in terms of the number of foreign languages, it seems reasonable to omit this respondent data variable from further analysis. Indeed, Pearson’s correlation coefficient between nationality and the number of languages is 0.905 (*p* < 0.001), and therefore it was decided to exclude the sociodemographic variable “Number of languages” from further analysis.

## 3. Results

In the core section, students evaluated 19 statements (see below) connected to the use of digital media and their relation to L2 acquisition. The usual analysis of a full group of statements can be problematic to interpret. Therefore, whether the reduction of dimensions makes sense and is valid was checked.

First, the correlation matrix formed from the output set was checked. Green indicates values in the ranges (−1; 0.3> and <0.3; 1), orange indicates (−0.3; −0.15> and <0.15; 0.3), and red indicates (−0.15; 0.15). The details are in Table 2. 

Based on the graphical analysis of the correlation matrix, one can conclude that the variables are not redundant and data reduction analysis will not yield a valid result here. Indeed, the standard Kaiser–Meyer–Olkin index here does not exceed the required threshold of 0.5 (specifically 0.496); therefore, dimensionality reduction will not have a meaningful effect.

Given the above, it was decided not to reduce factors, which implies a full-scale analysis.

### 3.1. Quantitative Analysis of the Results

The following analysis is based on 19 statements that were rated on a 5-point Likert scale (strongly disagree, disagree, undecided, agree, strongly agree):

S1. I like learning foreign languages.
S2. I prefer printed learning materials to electronic ones.
S3. I prefer learning with a teacher in a classroom to online learning.
S4. I miss meeting my classmates while I have online classes.
S5. Online classes are more convenient for me than learning in a classroom.
S6. Online communication with the teacher via email and eLearning platforms is better for me than physical communication at school.
S7. Online classes are more efficient for me than traditional classes with a teacher.
S8. I think that foreign languages will be useful in my future career.
S9. Electronic texts are better for me than printed books.
S10. I think that online language learning is more effective than traditional learning in a classroom.
S11. I am not worried and I trust myself when I study foreign languages online.
S12. I think that my language teacher is well prepared to give instructions online.
S13. I am OK with having my camera turned on during the online foreign language course.
S14. I think that online language teaching is very interactive.
S15. I feel I am improving my language skills while studying online.
S16. I enjoy using a virtual foreign language learning environment (e.g., MS Teams, Blackboard, etc.).
S17. I receive enough feedback online on my language learning from the teacher.
S18. I prefer online language teaching to face-to-face teaching.
S19. I think that we spend too much time in front of the computer.

Respondents’ answers were recorded on a scale of 1 to 5, and then the mean for each question was calculated. Treating the results as quantitative data allows for the determination of the degree of acceptance of the statements, with a mean score of 3 indicating a neutral opinion. The highest acceptance was observed for statement 8, while the lowest—for statement 7. A full breakdown of means is contained in Table 3.

Polish students were significantly more likely to agree with statement 17 and strongly agree with statements 4, 8, and 12. These last three statements and statement 19 are the items with the highest percentage of strongly-agree responses in the Polish group. Overall, except for statements 9 and 11, Czechs were less likely to indicate an extremely positive response. Only in the case of 3 statements (1, 5, and 11), Polish students were noticeably more likely (i.e., a difference of more than 1 pp) to be unable to state their views. The statements with which respondents disagreed most frequently were S6, S7, S9, S10, and S18. No apparent prevalence of negative or positive attitudes was recorded for statements 13, 14, and 16. For details see Table 4.

Polish students from age group 1 were more likely to agree with statement 3, while Czech students were more likely to strongly disagree with the statement and gave neutral responses. In the case of the age group 2, it was noted that Polish students were more likely than their Czech peers to express agreement in the case of statements 8, 12, and 17, with noticeable indecisiveness of Czech students observed for statement 17, as evidenced by the number of neutral answers. Czech respondents in age group 3 were more likely than their Polish peers to agree with statements 7 and 9, while Polish respondents were more likely to agree with statements 4, 8, 12, and 19. For details see Table 5.

In the case of the analysis of the students surveyed by gender, it was found that Czech male students were more likely than Polish male students to agree with statements 7 and 10, with Poles more likely than Czechs to give negative answers in both cases, Polish students more likely to agree with statements 4, 8, 12, and 17, and Czech students much more likely to avoid giving a clear response to those statements. Polish female students were more likely to agree with statements 8, 12, and 17 than their female peers from the Czech Republic. In the case of statement 8, the difference was due to greater indecision of Czech female students, while in statements 12 and 17, Czech female students were more likely to give negative responses. For details see Table 6.

Polish students who spent 6 to 12 h a day in front of the computer were more likely to agree with statements 2, 4, 8, 12, and 17 than their Czech peers. In the case of statement 2, Czech students more often than their Polish peers gave negative responses and avoided giving a clear answer, while in the case of statements 4, 8, 12, and 17, there was a significantly higher number of neutral indications. Czech students who spent 6 to 12 h a day in front of the computer were more likely to agree with statements 7, 10, and 18, whereas their Polish peers were more likely to indicate negative answers. Polish students who spent 12 to 16 h a day in front of the computer were more likely to agree with statement 12, while Czech students were more likely to give a neutral or negative answer. It was noted that in the case of students spending between 2 and 6 h a day in front of the computer, Czech respondents were more likely to disagree with statement 13 or give a neutral answer. For details see Table 7.

### 3.2. Qualitative Analysis of the Results

Qualitative analysis of the data was used to study the responses in order to obtain a complementary picture of students’ attitudes towards online language learning. Based on the content of the questionnaire, the following statements were selected: six statements that served as students’ initial attitudes toward: foreign language learning: in general and online, contact with other students, using online contact programs, and computer use. And seven statements that were used to compare online learning and face-to-face learning.

It was verified whether students’ initial attitudes significantly influenced their response choices for other statements (i.e., whether differences in responses were statistically significant). The following statements were used as initial variables here:

S1 as an attitude towards language learning (in general, whether the person likes or dislikes learning): Indeed, students who liked learning foreign languages were more likely to say that this skill would be useful in their future careers (S8). A similar relationship is observed for statement S11: higher self-esteem is evident in terms of self-confidence when learning foreign languages online. People who did not like learning foreign languages or were neutral in replying to this statement were more likely to disagree with statement S11.

S4 as an attitude towards contact with other students (or rather the lack of contact that resulted from distance learning): Respondents who did not have an opinion about meeting other students in online classes were more likely to be neutral about the teacher’s preparation to deliver online instruction (S12) and about the preference for face-to-face teaching over remote teaching (S3), while those who strongly agreed with statement S4 were more likely to strongly agree with statement S3. Respondents who missed contact with other students were less likely to acknowledge the superiority of online communication with the teacher over face-to-face communication at the university (S6), and more likely to disagree with the statement that online classes are more effective (S7), efficient (S10), or convenient (S5). As the acceptance of statement S4 increases, the percentage of people who disagree with statement S18 increases, and at the same time the percentage of people who agree with the statement decreases. Respondents who strongly identified with statement S4 were more likely to indicate a strongly-agree response to statement S19. This percentage is much lower for those who have a neutral opinion about meeting other students.

S8 as an attitude towards the validity of learning foreign languages (in the context of their usefulness in the future): Those who were undecided about the usefulness of language learning were more likely to disagree with statement S1 and more likely to agree with statement S12. These respondents were also more likely to have difficulty stating their opinion on statement S16.

S14 as an attitude towards online learning (here: determining the degree of interactivity): Respondents who disagreed with the statement that remote learning is interactive were also more likely to disagree with a statement that online learning is more effective (S10). On the other hand, those who confirmed that remote classes were more interactive were much more likely to strongly agree that their foreign language teacher was well prepared to provide online instruction (S12) and gave them feedback on the appropriate extent (S17), were more likely to feel that their language skills improved while learning online (S15), and were more likely to like using a virtual language learning environment (S16). These individuals were also more likely to prefer online classes over face-to-face classes (S18).

S16 as an attitude towards the use of online communication programs (especially using them in remote language learning): It was noted that respondents who disliked using virtual language learning environments, such as MS Teams, were far more likely to indicate that they preferred classroom learning to online language learning. Respondents who liked to use online language learning software were more likely to agree with the statement that communication with a teacher online is better than face-to-face communication in school or university. Respondents who did not like using a virtual environment for language learning were most likely to indicate that online classes are not more effective than face-to-face classes, and more likely to disagree that online classes are more effective than face-to-face classes.

Regardless of whether respondents liked using online language learning software or not, they claimed that language skills would be useful in their future careers. Those who enjoy using a virtual language learning environment were significantly more likely to admit that they were confident about their skills in language learning. Respondents who did not like using a virtual language learning environment were more likely to think that remote language learning is very interactive, while respondents who liked learning via online programs were far more likely to agree with the statement that they felt they were improving their language skills while learning remotely. Students who disliked using virtual language learning environments were more likely to indicate that they definitely did not receive enough feedback on their learning progress. Furthermore, respondents who did not like using virtual language learning programs were more likely to strongly disagree with the statement that they preferred learning foreign languages remotely to face-to-face learning.

S19 as an attitude towards time spent in front of the computer: Students who agreed with statement S19 were less likely to disagree with the statement about lack of contact with peers in online classes, but more likely to agree with statement S4. Respondents who had a neutral opinion towards statement S19 were apparently less likely to strongly agree with statement S12.

Contradictory statements were used for analysis: S2 vs. S9 and S3 vs. S18.

Based on the comparison of the type of materials, students apparently preferred the paper version (Table 8 below), while in terms of the type of learning, respondents preferred classroom learning (Table 9 below).

## 4. Discussion

Overall, to obtain answers to the research questions, the results of this study reveal that university students learning a foreign language, in this case English, prefer any kind of face-to-face learning to online (remote) learning, which they had to experience for the whole academic year during the COVID-19 pandemic. This is in fact in line with the findings of [25] who in their research study show that the exclusive use of various eLearning tools in the post-COVID period was not preferred by more than 50% of the participants of their research, while implementation of blended learning (which is a combination of face-to-face learning and online learning) was rejected by less than a half of the participants. Similar results were described by [26] who reported in his study that EFL learners’ satisfaction with online learning was relatively low, i.e., less than half of the respondents were satisfied with online learning, while only 14% of them did not favor online learning, and nearly a half (43%) of the respondents did not support continuing with online education. Furthermore, if students in the present study liked learning a foreign language virtually, they considered this environment convenient, but less effective. Refs. [27,28] expand that students generally support and appreciate online learning less and they generally agree that their motivation could even decrease when they have to study online.

In addition, as far as the medium of L2 acquisition is concerned, students prefer print/paper learning materials to electronic ones, which might be connected to the fact that students are used to learning new words and phrases by highlighting them in the text and having them at hand when needed. This has also been confirmed by [29], who found out that digital teaching materials cannot be adequate and sufficient for note-taking and they do not provide material for the students to use these more actively. Therefore, printed materials are generally preferred for a better understanding of the written text as any kind of motion and activity in learning leads to better study results. Movement, writing, and making notes are helpful cognitive strategies to strengthen memory, retrieval of information, and retention [30]. Another fact might be that they do not want to spend time in front of the computer, which was also pointed out as a drawback of online learning in this current study. The screen time increase must be further researched as it is potentially the major drawback of any kind of eLearning and the users realize its negative impact on their well-being. However, generally, there is a trend to use a hybrid mode of learning materials, i.e., both electronic and paper since the first are more ecological and usually less costly, as well as up-to-date, and the latter ones might be of better quality and all the materials are not always online (cf. [31,32]).

Moreover, the drawback of the electronic mode of learning a foreign language was also social distancing. As research shows, students felt frustrated about the lack of social contact (cf. [25]). Ref. [33], for instance, claim that the social distancing restrictions imposed on students and teachers stopped the natural development of various crucial academic for the development of academic languages, such as book reading with peers and various kinds of group discussions. Furthermore, students did not feel very comfortable with their cameras on, which might also have been connected to their lower proficiency and self-confidence in language learning (cf. [25]).

As far as the differences between the Czech and Polish students are concerned, the findings reveal that the Czech students were more neutral in their responses, especially about the sufficient feedback from the teacher. As [25] maintain, the reason is that the response in the online environment is delayed.

Although students generally agree with the fact that the teachers are almost always well-prepared for the classes online, ref. [34] report that all the possibilities of traditional classes, namely, the communication opportunity, are not transferred to online classes as it is almost impossible. The Internet does not allow teachers to provide a teaching presence that can be considered as a very important aspect of any teaching process as a crucial catalyst of the traditional teaching process. Online learning, on the other hand, overloads students with a lot of various digital assignments and they are not able to communicate sufficiently, which is the key way how to improve their professional communicative competence.

## 5. Conclusions

The main limitations of the research are caused by the relatively geographically small scale of the research as it was conducted only in two neighboring central European countries. It will be necessary to replicate the research on a much larger geographical scale to obtain more reliable results; however, the research conducted can be sufficient statistically and the results yielded are replicable. Future research should focus on the verification of our findings on a larger geographical scale and with a larger research sample, it should also take into account other more sophisticated tools for eLearning that utilize artificial intelligence and other tools, such as big data and deep learning. In addition, the concept of teachers’ technological pedagogical content knowledge (TPACK) skills will have to be reviewed [35].

The implications of the findings are far-reaching for both the educators and curricula makers, but also for designers of mobile apps and various communication platforms for education. The findings clearly show that it is crucial for the educators to use various mobile and online tools carefully as they are not perceived very positively by the users if there is no adequate utilization of them. The students are very sensitive to their implementation as they do not perceive them as being very beneficial compared to face-to-face classes. The conducted research clearly shows that the respondents realized that online classes cannot substitute standard classes when they can meet the tutor and also other students, have a discussion, and various class activities. The respondents are fully aware of this drawback of online classes, moreover, they also expressed their preference for print media over digital media for L2 acquisition. It is in line with other research which is still very limited.

Therefore, this pilot research study is an impetus for further studies that must be conducted to clearly highlight the potential but also drawbacks of online education. Further studies should focus on practical advice on how to use various digital tools more efficiently so that the users can benefit from them. There is too much theoretical research or practical research with theoretical findings only; however, it is crucial to provide educators very clear strategies on how to use online learning to its fullest potential, not forgetting its pitfalls.

## Figures and Tables

**Table 1:** Characterization of respondents.

Respondent Data	CZ (N)	CZ (%)	PL (N)	PL (%)	Total (N)	Total (%)
NATIONALITY						
CZ					100	48.3%
PL					107	51.7%
AGE						
A1	14	14.0%	30	28.0%	44	21.2%
A2	47	47.0%	32	29.9%	79	38.2%
A3	19	19.0%	34	31.8%	53	25.6%
A4	20	20.0%	11	10.3%	31	15.0%
GENDER						
Woman	46	46.0%	52	48.6%	98	47.3%
Man	54	54.0%	55	51.4%	109	52.7%
FOREIGN LANGUAGE						
English	100	100.0%	107	100.0%	207	100.0%
German	5	5.0%	45	42.1%	50	24.2%
Spanish	1	1.0%	41	38.3%	42	20.3%
Other	2	2.0%	20	18.7%	22	10.6%
One language	92	92.0%	2	1.9%	94	45.4%
Two and more	8	8.0%	105	98.1%	113	54.6%
TIME SPENT IN FRONT OF THE COMPUTER						
up to 2 h a day	3	3.0%	1	0.9%	4	1.9%
between 2 and 6 h a day	17	17.0%	13	12.1%	30	14.5%
between 6 and 12 h a day	59	59.0%	65	60.8%	124	59.9%
between 12 and 16 h a day	15	15.0%	23	21.5%	38	18.4%
more than 16 h a day	6	6.0%	5	4.7%	11	5.3%
SKILLS GOOD FOR ONLINE PRACTICE						
grammar	49	49.0%	52	48.6%	101	48.8%
listening	67	67.0%	59	55.1%	126	60.9%
pronunciation	28	28.0%	24	22.4%	52	25.1%
reading	47	47.0%	62	57.9%	109	52.7%
speaking	50	50.0%	55	51.4%	105	50.7%
writing	37	37.0%	54	50.5%	91	44.0%
vocabulary	66	66.0%	68	63.6%	134	64.7%
NUMBER OF SKILLS INDICATED						
one	8	8.0%	4	3.7%	12	5.8%
two	19	19.0%	22	20.6%	41	19.8%
three	25	25.0%	31	29.0%	56	27.1%
four	26	26.0%	30	28.0%	56	27.1%
five	17	17.0%	13	12.2%	30	14.5%
six	1	1.0%	1	0.9%	2	1.0%
seven	4	4.0%	6	5.6%	10	4.8%

**Table 2:** Graphical interpretation of the correlation matrix.

	S1	S2	S3	S4	S5	S6	S7	S8	S9	S10	S11	S12	S13	S14	S15	S16	S17	S18	S19
S1																			
S2																			
S3																			
S4																			
S5																			
S6																			
S7																			
S8																			
S9																			
S10																			
S11																			
S12																			
S13																			
S14																			
S15																			
S16																			
S17																			
S18																			
S19																			

Green indicates values in the ranges (−1; 0.3> and <0.3; 1), orange indicates (−0.3; −0.15> and <0.15; 0.3), and red indicates (−0.15; 0.15).

**Table 3:** Recorded response mean.

Statement	Recoded Mean
S1	3.95
S2	3.53
S3	3.80
S4	4.11
S5	3.28
S6	2.44
S7	2.35
S8	4.59
S9	2.49
S10	2.37
S11	3.21
S12	4.19
S13	2.77
S14	3.03
S15	3.28
S16	2.95
S17	3.60
S18	2.40
S19	4.17

**Table 4:** Distribution of answers by nationality and significance of differences.

Statement	Nationality	Strongly Disagree	Disagree	Undecided	Agree	Strongly Agree	Significance of Differences
S1	CZ	2.0%	10.0%	9.0%	58.0%	21.0%	*p* = 0.265
	PL	1.9%	2.8%	12.1%	57.0%	26.2%	
S2	CZ	2.0%	20.0%	27.0%	34.0%	17.0%	*p* = 0.482
	PL	3.7%	15.0%	24.3%	30.8%	26.2%	
S3	CZ	2.0%	9.0%	27.0%	43.0%	19.0%	*p* = 0.502
	PL	0.9%	5.6%	24.3%	40.2%	29.0%	
S4	**CZ**	**2.0%**	**11.0%**	**15.0%**	**38.0%**	**34.0%**	***p* = 0.015**
	**PL**	**0.0%**	**9.3%**	**6.5%**	**29.9%**	**54.2%**	
S5	CZ	3.0%	28.0%	22.0%	38.0%	9.0%	*p* = 0.427
	PL	5.6%	18.7%	26.2%	36.4%	13.1%	
S6	CZ	11.0%	46.0%	24.0%	18.0%	1.0%	*p* = 0.602
	PL	19.6%	42.1%	20.6%	16.8%	0.9%	
S7	CZ	13.0%	35.0%	40.0%	11.0%	1.0%	*p* = 0.088
	PL	21.5%	43.9%	28.0%	6.5%	0.0%	
S8	**CZ**	**0.0%**	**1.0%**	**8.0%**	**37.0%**	**54.0%**	***p* = 0.003**
	**PL**	**0.0%**	**0.9%**	**2.8%**	**18.7%**	**77.6%**	
S9	CZ	12.0%	40.0%	28.0%	13.0%	7.0%	*p* = 0.304
	PL	21.5%	38.3%	25.2%	12.1%	2.8%	
S10	CZ	10.0%	43.0%	35.0%	12.0%	0.0%	*p* = 0.105
	PL	15.9%	47.7%	31.8%	3.7%	0.9%	
S11	CZ	3.0%	27.0%	14.0%	50.0%	6.0%	*p* = 0.149
	PL	3.7%	25.2%	28.0%	39.3%	3.7%	
S12	**CZ**	**2.0%**	**2.0%**	**21.0%**	**50.0%**	**25.0%**	***p* < 0.001**
	**PL**	**0.0%**	**0.0%**	**1.9%**	**53.3%**	**44.9%**	
S13	CZ	17.0%	36.0%	14.0%	28.0%	5.0%	*p* = 0.874
	PL	13.1%	32.7%	15.0%	33.6%	5.6%	
S14	CZ	5.0%	20.0%	42.0%	30.0%	3.0%	*p* = 0.071
	PL	6.5%	30.8%	24.3%	32.7%	5.6%	
S15	CZ	7.0%	13.0%	34.0%	45.0%	1.0%	*p* = 0.502
	PL	1.9%	14.0%	32.7%	50.5%	0.9%	
S16	CZ	6.0%	28.0%	37.0%	27.0%	2.0%	*p* = 0.926
	PL	6.5%	26.2%	32.7%	31.8%	2.8%	
S17	**CZ**	**2.0%**	**13.0%**	**35.0%**	**45.0%**	**5.0%**	***p* < 0.001**
	**PL**	**0.9%**	**4.7%**	**14.0%**	**72.9%**	**7.5%**	
S18	CZ	10.0%	48.0%	28.0%	12.0%	2.0%	*p* = 0.147
	PL	22.4%	40.2%	24.3%	9.3%	3.7%	
S19	CZ	4.0%	10.0%	8.0%	38.0%	40.0%	*p* = 0.214
	PL	0.9%	5.6%	4.7%	36.4%	52.3%	

**Table 5:** Analysis of the prevalence of agreement with statements by nationality and age.

Statement	Age Group	Prevalence of Agreement	Significance of Differences
S3	A1	PL > CZ	*p* = 0.049
S4	A3	PL > CZ	*p* = 0.013
S7	A3	CZ > PL	*p* = 0.013
S8	A2	PL > CZ	*p* = 0.042
	A3	PL > CZ	*p* = 0.004
S9	A3	CZ > PL	*p* = 0.007
S12	A2	PL > CZ	*p* = 0.012
	A3	PL > CZ	*p* = 0.014
S17	A2	PL > CZ	*p* = 0.002
S19	A3	PL > CZ	*p* = 0.003

**Table 6:** Analysis of the prevalence of agreement with statements by nationality and gender.

Statement	Gender	Prevalence of Agreement	Significance of Differences
S4	M	PL > CZ	*p* = 0.011
S7	M	CZ > PL	*p* = 0.006
S8	F	PL > CZ	*p* = 0.009
	M	PL > CZ	*p* = 0.016
S10	M	CZ > PL	*p* = 0.030
S12	F	PL > CZ	*p* = 0.003
	M	PL > CZ	*p* = 0.001
S17	F	PL > CZ	*p* = 0.003
	M	PL > CZ	*p* = 0.004

**Table 7:** Analysis of the prevalence of agreement with statements by nationality and time spent in front of the computer.

Statement	Time Spent in Front of the Computer	Prevalence of Agreement	Significance of Differences
S2	6–12 h	PL > CZ	*p* = 0.021
S4	6–12 h	PL > CZ	*p* = 0.009
S7	6–12 h	CZ > PL	*p* = 0.010
S8	6–12 h	PL > CZ	*p* = 0.005
S10	6–12 h	CZ > PL	*p* = 0.003
S12	6–12 h	PL > CZ	*p* < 0.001
	12–16 h	PL > CZ	*p* = 0.045
S13	2–6 h	PL > CZ	*p* = 0.037
S17	6–12 h	PL > CZ	*p* < 0.001
S18	6–12 h	CZ > PL	*p* = 0.021

**Table 8:** Comparison of opinions on paper and electronic materials.

	Strongly Disagree	Disagree	Undecided	Agree	Strongly Agree
Printed > Electronic	2.9%	17.4%	25.6%	32.4%	21.7%
Electronic > Printed	16.9%	39.1%	26.6%	12.6%	4.8%

Source: author’s own elaboration.

**Table 9:** Comparison of opinions about face-to-face and remote learning.

	Strongly Disagree	Disagree	Undecided	Agree	Strongly Agree
Stationary > Online	1.4%	7.2%	25.6%	41.5%	24.2%
Online > Stationary	16.4%	44.0%	26.1%	10.6%	2.9%

## Data Availability

All data received are present in the manuscript.

## References

[1] Huang X. (2018). Improving communicative competence through synchronous communication in computer-supported collaborative learning environments: A systematic review. Educ. Sci..

[2] Chen M.L. (2018). A data-driven critical review of second language acquisition in the past 30 years. Publications.

[3] Klimova B., Zamborova K. (2020). Use of mobile applications in developing reading comprehension in second language acquisition—A review study. Educ. Sci..

[4] Klimova B., Pikhart M. (2021). New advances in second language acquisition methodology in higher education. Educ. Sci..

[5] Mystakidis S., Berki E., Valtanen J.P. (2021). Deep and meaningful e-learning with social virtual reality environments in higher education: A systematic literature review. Appl. Sci..

[6] Den E.K., Rowe S., Boyd W., Lloyd D. (2012). Using web 2.0 technologies for collaborative learning in distance education—Case studies from an Australian university. Future Internet.

[7] Bujang S.D.A., Selamat A., Krejcar O., Maresova P., Nguyen N.T. (2020). Digital learning demand for future education 4.0—Case studies at Malaysia education institutions. Informatics.

[8] Pikhart M., Klímová B. (2020). eLearning 4.0 as a sustainability strategy for generation Z language learners: Applied linguistics of second language acquisition in younger adults. Societies.

[9] Verma C., Illés Z., Stoffová V., Bakonyi V. (2020). Opinion prediction of Hungarian students for real-time e-learning systems: A futuristic sustainable technology-based solution. Sustainability.

[10] Salmon G., Wright P. (2014). Transforming future teaching through ‘Carpe Diem’ learning design. Educ. Sci..

[11] Liyanagunawardena T.R. (2015). Massive open online courses. Humanities.

[12] Sun P.C., Finger G., Liu Z.L. (2014). Mapping the evolution of elearning from 1977–2005 to inform understandings of elearning historical trends. Educ. Sci..

[13] Becker B.A., Eube C. (2018). Open innovation concept: Integrating universities and business in digital age. J. Open Innov. Technol. Mark. Complex..

[14] Antoniou M., Wright S.M. (2017). Uncovering the Mechanisms Responsible for Why Language Learning May Promote Healthy Cognitive Aging. Front. Psychol..

[15] Liu M., Zheng Y., Ma X., Wei Y. (2020). Sustaining multilingualism in Chinese universities: Uzbekistani students’ demotivation while learning Chinese. Sustainability.

[16] Teeter J.L. (2017). Improving motivation to learn English in Japan with a self-study shadowing application. Languages.

[17] Martínez-Roget F., Freire Esparís P., Vázquez-Rozas E. (2020). University student satisfaction and skill acquisition: Evidence from the undergraduate dissertation. Educ. Sci..

[18] Hahlin C.R., Granfeldt J. (2021). Strengthening L3 French motivation: The differential impact of vision-enhancing activities. Languages.

[19] Isbell D.R., Rawal H., Oh R., Loewen S. (2017). Narrative perspectives on self-directed foreign language learning in a computer- and mobile-assisted language learning context. Languages.

[20] Almarzooq Z.I., Lopes M., Kochar A. (2020). Virtual learning during the COVID-19 pandemic: A disruptive technology in graduate medical education. J. Am. Coll. Cardiol..

[21] Gohiya P., Gohiya A. E-Learning during COVID-19 Pandemic. https://www.researchsquare.com/article/rs-29575/v1.

[22] Sindiani A.M., Obeidat N., Alshdaifat E., Elsalem L., Alwani M.M., Rawashdeh H., Fares A.S., Alalawne T., Tawalbeh L.I. (2021). Distance education during the COVID-19 outbreak: A cross-sectional study among medical students in North of Jordan. Ann. Med. Surg..

[23] Dhawan S. (2020). Online learning: A panacea in the time of COVID-19 crisis. J. Educ. Technol. Syst..

[24] Anwar K., Adnan M. (2020). Online learning amid the COVID-19 pandemic: Students perspectives. J. Pedagog. Res..

[25] Maican M.A., Cocoradă E. (2021). Online foreign language learning in higher education and its correlates during the COVID-19 pandemic. Sustainability.

[26] Mahyoob M. (2020). Challenges of e-learning during the COVID-19 pandemic experienced by EFL learners. Arab World Engl. J..

[27] Consilz T. (2020). The impact of COVID-19 on student motivation, community of inquiry and learning performance. Asian Educ. Dev. Stud..

[28] Meeter M., Bele T., den Hartogh C., Bekker T. (2020). College Students’ Motivation and Study Results after COVID-19 Stay-at-Home Orders. https://www.researchgate.net/publication/346172498_College_students%27_motivation_and_study_results_after_COVID-19_stay-at-home_orders.

[29] Vilar P., Zabukovec V. Using e-materials for study: Students’ perceptions vs. perceptions of academic librarians and teachers. Proceedings of the ISIC, the Information Behaviour Conference.

[30] Jensen E.P. (2020). Brain-Based Learning: Teaching the Way Students Really Learn.

[31] Liu Z. (2006). Print vs. electronic resources: A study of user perceptions, preferences, and use. Inf. Process. Manag..

[32] Shahzad S.K., Hussain J., Sadaf N., Sarwat S., Ghani U., Saleem R. (2020). Impact of virtual teaching on ESL learners’ attitudes under COVID-19 circumstances at post graduate level in Pakistan. Engl. Lang. Teach..

[33] Li G., Dobrin-De Grâce R., Sun Z., Haslip M., Burchell D., Dexter J.R., Chen X. (2021). Promoting Second Language Learning during the COVID-19 Pandemic: Parents’ and Teachers’ Coping Strategies. https://rsc-src.ca/fr/voix-de-la-src/promoting-second-language-learning-during-covid-19-pandemic-parents%E2%80%99-and-teachers%E2%80%99.

[34] Poluekhtova I., Vikhrova O., Vartanova E. (2020). Effectiveness of online education for the professional training of journalists: Students’ distance learning during the COVID-19 pandemic. Psychol. Russ. State Art.

[35] Md Yunus M., Ang W.S., Hashim H. (2021). Factors affecting teaching English as a second language (TESL) postgraduate students’ behavioural intention for online learning during the COVID-19 pandemic. Sustainability.

